# A Protein Aggregation Inhibitor, Leuco-Methylthioninium Bis(Hydromethanesulfonate), Decreases α-Synuclein Inclusions in a Transgenic Mouse Model of Synucleinopathy

**DOI:** 10.3389/fnmol.2017.00447

**Published:** 2018-01-10

**Authors:** Karima Schwab, Silke Frahm, David Horsley, Janet E. Rickard, Valeria Melis, Elizabeth A. Goatman, Mandy Magbagbeolu, Morag Douglas, Michael G. Leith, Thomas C. Baddeley, John M. D. Storey, Gernot Riedel, Claude M. Wischik, Charles R. Harrington, Franz Theuring

**Affiliations:** ^1^Institute of Pharmacology, Charite – Universitätsmedizin Berlin, Berlin, Germany; ^2^School of Medicine, Medical Sciences and Nutrition, University of Aberdeen, Aberdeen, United Kingdom; ^3^Department of Chemistry, University of Aberdeen, Aberdeen, United Kingdom; ^4^TauRx Therapeutics Ltd., Singapore, Singapore

**Keywords:** aggregation inhibitor, leucomethylthioninium, mouse model, Parkinson’s disease, α-synuclein, synucleinopathy

## Abstract

α-Synuclein (α-Syn) aggregation is a pathological feature of synucleinopathies, neurodegenerative disorders that include Parkinson’s disease (PD). We have tested whether *N,N,N′,N′*-tetramethyl-10*H*-phenothiazine-3,7-diaminium bis(hydromethanesulfonate) (leuco-methylthioninium bis(hydromethanesulfonate); LMTM), a tau aggregation inhibitor, affects α-Syn aggregation *in vitro* and *in vivo*. Both cellular and transgenic models in which the expression of full-length human α-Syn (h-α-Syn) fused with a signal sequence peptide to promote α-Syn aggregation were used. Aggregated α-Syn was observed following differentiation of N1E-115 neuroblastoma cells transfected with h-α-Syn. The appearance of aggregated α-Syn was inhibited by LMTM, with an EC_50_ of 1.1 μM, with minimal effect on h-α-Syn mRNA levels being observed. Two independent lines of mice (L58 and L62) transgenic for the same fusion protein accumulated neuronal h-α-Syn that, with aging, developed into fibrillary inclusions characterized by both resistance to proteinase K (PK)-cleavage and their ability to bind thiazin red. There was a significant decrease in α-Syn-positive neurons in multiple brain regions following oral treatment of male and female mice with LMTM administered daily for 6 weeks at 5 and 15 mg MT/kg. The early aggregates of α-Syn and the late-stage fibrillar inclusions were both susceptible to inhibition by LMTM, a treatment that also resulted in the rescue of movement and anxiety-related traits in these mice. The results suggest that LMTM may provide a potential disease modification therapy in PD and other synucleinopathies through the inhibition of α-Syn aggregation.

## Introduction

Protein conformational disorders are a group of diseases which evolve due to protein misfolding leading to the formation of aggregated structures (Carrell and Gooptu, 1998; Uversky and Fink, 2004; Lashuel et al., 2013). PD and dementia with Lewy bodies are the most common disorders associated with synuclein pathology and are characterized by intra-neuronal deposits of α-Syn fibrils most prominently found in midbrain regions and cerebral cortex (Uversky, 2003). This pathology spreads progressively from medulla oblongata through midbrain to neocortex (Braak et al., 2003). The monomeric α-Syn is a protein of 140 amino acids that undergoes a conformational change associated with the formation of dimers and oligomers and, ultimately, pre-fibrils and insoluble fibrils (Uversky and Fink, 2004; Lashuel et al., 2013). Mutations in the α-synuclein gene (*SNCA*), e.g., A53P, A53T, and E46K, promote self-oligomerisation and are associated with dominantly inherited PD (Uversky and Fink, 2004; Lashuel et al., 2013).

We have developed two transgenic mouse models of synucleinopathy, h-α-SynL58 and h-α-SynL62, termed L58 and L62, respectively. In both, full-length h-α-Syn fused with an N-terminal signal sequence peptide, is expressed under the control of the *Thy1*-promotor. The signal sequence peptide serves as a membrane targeting sequence that directs proteins to the endoplasmic reticulum (Partridge et al., 1999; Harrington et al., 2015; Melis et al., 2015b). A detailed characterisation of L58 and L62 is reported separately (Frahm et al., 2017). There is intra-neuronal expression of h-α-Syn in both neocortex and archicortex in these mouse lines, and both are associated with a behavioral hypoactivity state.

Although levodopa remains the most effective treatment of PD, side effects such as motor fluctuations and dyskinesia limit its long-term use. Alternative approaches targeting α-Syn include enhancement of autophagy, modulation of phosphorylation, and slowing or ameliorating the production, deposition and aggregation of pathological proteins (Dehay et al., 2015). This presents several new therapeutic targets, including active or passive immunization these have been summarized by Velayudhan et al. (2017). Interference with anti-parallel folding of α-Syn and/or disaggregation of existing oligomers are of particular interest. Polyphenols and peptides containing mutated segments of the hydrophobic central region of α-Syn, and tyrosine or tryptophan-rich β-hairpins, have been reported to inhibit synuclein aggregation (for examples, see Sivanesam et al., 2015). Inhibition of the cytotoxic A11 epitopes of synuclein by triphenylmethane dyes such as Coomassie Brilliant Blue R disrupts existing fibrils and reduces fibrillisation (Ahsan et al., 2017), but none of the above molecules has been tested in clinical trials. Methylthioninium chloride (MTC) and leuco-methylthioninium bis(hydromethanesulfonate) (LMTM) are effective as protein aggregation inhibitors for both tau and α-Syn *in vitro* (Wischik et al., 1996; Taniguchi et al., 2005; Masuda et al., 2006; Harrington et al., 2015) and are capable of rescuing behavioral deficits and ameliorating pathology in transgenic mouse models of tau protein aggregation (Melis et al., 2015a). Both forms of the methylthioninium (MT) moiety have been tested clinically in Alzheimer’s disease in Phase 2 and 3 clinical trials (Wischik et al., 2015; Gauthier et al., 2016; Wilcock et al., 2018) and have been found to have potential efficacy as monotherapy. LMTM is a stable reduced form of the MT moiety, which is better absorbed than MTC over a wide dosing range.

We now report that LMTM inhibits α-Syn aggregation in N1E-115 neuroblastoma cells expressing human α-Syn and in cortical neurons of transgenic L58 and L62 mice. Amelioration of h-α-Syn pathology was associated with normalization of behavioral deficits in L62 mice.

## Materials and Methods

### LMTM and Antibodies

*N,N,N′,N′*-tetramethyl-10*H*-phenothiazine-3,7-diaminium bis (methanesulfonate) (leuco-methylthioninium bis(hydro- methanesulfonate); LMTM) was supplied by TauRx Therapeutics Ltd., Singapore.

A list of the α-Syn antibodies used in this study, their epitopes and source, is given in **Table 1**.

**Table 1:** Description of α-Syn antibodies.

Antibody	Species/Class	Immunogen	Epitope (specificity)	Source (catalog number)
mAb 42	Mouse IgG1	rat-α-Syn	15–123	BD Biosciences, Oxford, United Kingdom (610787)
mAb 204	Mouse IgG2a	h-α-Syn	95∼109^∗^	Santa Cruz Biotechnology, Dallas, TX, United States (sc-32280)
mAb 211	Mouse IgG1	h-α-Syn 121–125 peptide	121∼125	Santa Cruz Biotechnology (sc-12767)
mAb 559	Mouse IgG1	h-α-Syn	109∼140^∗^	AMS Biotechnology, Abingdon, United Kingdom
mAb 874	Mouse IgG1	h-α-Syn	109∼140^∗^	AMS Biotechnology
mAb 3H2897	Mouse IgG1	h-α-Syn	1–140	Santa Cruz Biotechnology (sc-69977)
mAb D37A6	Rabbit IgG	mouse-α-Syn 100–110 peptide	100∼110 (Mouse/rat-specific)	Cell Signaling Technology, Leiden, Netherlands (4179)

*^∗^Epitope mapping by immunoassay against recombinant and synthetic peptides in this study.*

### Cellular h-α-Syn Model

N1E-115 mouse neuroblastoma cells were modified to constitutively express full-length h-α-Syn fused with an N-terminal signal sequence peptide (SSFsyn) (DH60.21 clone; pcDNA3.1 vector). Cells were grown at 37°C in an atmosphere containing 5% CO_2_ in Dulbecco’s modified Eagle’s medium with 1% GlutaMAX I (1%), pyruvate and glucose (4.5 g/L) (Life Technologies, Paisley, United Kingdom) supplemented with 10% fetal calf serum (Sigma–Aldrich, Dorset, United Kingdom), penicillin (50 U/ml), streptomycin (50 μg/mL) and G418 (300 μg/mL; for maintenance of the plasmid). Transgene expression was induced by differentiation of the cells either by depletion of serum or by addition of either NGF (100 μg/mL) or dibuytryl cyclic AMP (db-cAMP; 1 mM) to serum-depleted medium. Cells were immunostained 72 h after induction, using anti-α-Syn antibodies as indicated. Goat anti-mouse IgG:FITC-conjugate (Sigma–Aldrich) was used as secondary antibody. Specimens were mounted with Vectashield Antifade Reagent (Vector Laboratories, Peterborough, United Kingdom) and examined by fluorescence microscopy (Axiomat; Carl Zeiss, Jena, Germany).

Transfected DH60.21 cells, in culture medium containing fetal calf serum (1%) and db-cAMP (1 mM), were treated with LMTM at five concentrations in triplicate. After 72 h incubation, medium was removed, cells were washed with PBS and collected in Laemmli SDS-PAGE sample buffer. Samples were separated by Tris-glycine SDS-PAGE, using a minigel system (BioRad Laboratories, Hemel Hempstead, United Kingdom). Proteins were transferred in *N*-cyclohexyl-3-aminopropanesulfonic acid (CAPS) buffer to polyvinylidene difluoride (PVDF) membrane and α-Syn detected on immunoblots with mAb 42 and enhanced chemiluminescence, and images captured using a Biospectrum 810 (UVP, Cambridge, United Kingdom). Samples from DH60.21 cells were separated by SDS-PAGE in duplicate side-by-side. One replicate was transferred to PVDF, and developed using mAb204. The other gel was fixed in acetic acid/ethanol and stained with silver, according to Nebrich et al. (2007). The stained gel was overlaid on the hyperfilm and spots, from silver-stained gel and corresponding to immunoreactive spots on film, were exicised from gel, digested with trypsin and measured with Q-Exactive Orbitrap. We detected no synuclein sequence in the 50-kDa band, whereas synuclein was identified in the 19-kDa synuclein band. The presence of a non-specific, non-synuclein band labeled with mAb 42 was used as an internal control for cell density in each replicate. The ratio of the intensities of the α-Syn band to the non-specific band were normalized to non-differentiated cells without drug treatment. The EC_50_ value was determined as the concentration at which this ratio is decreased by 50%.

Proteins were extracted from cells by sequential solubilisation using methods described by Masuda et al. (Masuda et al., 2006). DH60.21 cells were collected by centrifugation (350 × *g* for 5 min at 4°C), washed with PBS, and homogenized in Tris-HCl (30 mM; pH 7.5) with or without Triton X-100 (0.1%). The resultant supernatant fractions were centrifuged at 353,000 × *g* for 60 min at 4°C (Optima TLX Ultracentrifuge, Beckman Coulter, High Wycombe, United Kingdom). Samples were analyzed by SDS–PAGE and immunoblotting using mAb 42 and protein bands quantified by densitometry.

Cells treated with or without LMTM (2 μM) were prepared similarly for detection of α-Syn mRNA. RNA was extracted from frozen cell pellets using TRIzol^®^ (Invitrogen, Thermo Fisher Scientific, Waltham, MA, United States) and the concentration measured with a NanoDrop 1000 spectrophotometer (Thermo Fisher Scientific). RNA (5 μg) was treated with DNase (Applied Biosystems, Thermo Fisher Scientific), reverse transcribed with the iScript cDNA synthesis Kit (Bio-Rad, Hercules, CA, United States) and diluted to a final concentration of 2 ng/μL. Q-RT-PCR was carried out with Maxima SYBR Green (Applied Biosystems, Thermo Fisher Scientific). The ratio of h-α-Syn (forward primer: caaaaccaaggagggagtg, reverse primer: tcttctgggctactgctgtc) to GAPDH (forward primer: aacgaccccttcattgac, reverse primer: tccacgacatactcagcac) was calculated with the comparative Ct method and values were normalized to non-differentiated cells without drug treatment.

### Transgenic h-α-Syn Mice and Treatments

Transgenic mice are described in detail elsewhere (Frahm et al., 2017). L58 and L62 mice overexpress the same full-length h-α-Syn, described above for cells, fused to a membrane-targeting N-terminal signal sequence, under control of the mouse *Thy1*-promotor. Both male and female homozygous transgenic and wild-type C57BL/6J litters were housed in small colonies prior to and during experimentation (up to 6 per cage) in climatised holding rooms (20°C and 40% humidity) at 12 h light/dark cycle (lights on at 6 a.m.) with free access to food and water.

All animal experiments were performed in accordance with the European Communities Council Directive (63/2010/EU) and approved by the German Animal Research Ethics Committee of LAGESO (A0213/13).

Treatment cohorts aged 5–6 months were randomly assigned to groups based upon body weight and dosed with LMTM or vehicle via oral gavage, administered daily in the morning for 6 weeks (6 days per week, Monday–Saturday; 5 ml/kg body weight). Details of test cohorts are shown in **Tables 2, 3**. Treatment regime and dose selection was based on successful lowering of tau pathology in tau-transgenic mice (Melis et al., 2015a). LMTM was dissolved in vehicle (argon-sparged deionised water) and administered within 20 min of dissolution. The doses of LMTM are expressed in terms of free methylthioninium (MT) base per animal body weight (mg MT/kg).

**Table 2:** Treatment groups and cohort sizes (n) used for histopathology in L58 and L62.

Genotype	Age at beginning of study (months)	LMTM dose (mg MT/kg)	Gender	*n*
L58	5	0	M	5
			F	8
L58	5	5	M	7
			F	8
L58	5	15	M	7
			F	8
L62	5	0	M	7
			F	8
L62	5	5	M	6
			F	8
L62	5	15	M	7
			F	7

**Table 3:** Treatment groups and cohort sizes (*n*) used for behavioral assessment.

Genotype	Age at beginning of study (months)	LMTM dose (mg MT/kg)	Gender	*n*
Wild-type/L62	5.5	0	M	13/13
Wild-type/L62	5.5	1.5	M	14/14^∗^
Wild-type/L62	5.5	5.0	M	16/12

*^∗^No immunohistochemical analysis available for one mouse receiving 1.5 mg MT/kg.*

Twenty-four hours after the last drug administration, mice were sacrificed by cervical dislocation; brains were removed rapidly, the right hemisphere was fixed in formalin, wax embedded and processed for immunohistochemistry, as described previously (Melis et al., 2015a,b). The left hemisphere was frozen rapidly in liquid nitrogen and stored at -80°C for α-Syn and MT^+^ quantification, as described below.

### Quantification of α-Syn Proteins in Brain Tissue

Crushed frozen brain tissue was incubated for 45 min at room temperature in urea buffer (7 M urea, 2 M thiourea, 70 mM DTT, 25 mM Tris/HCl, 50 mM KCl, 3 mM EDTA, 2.9 mM benzamidine and 2.1 μM leupeptin) or at 4°C in Tris-buffer (30 mM; pH 7.5) and centrifuged for 45 min at 16,000 × *g*. Protein concentration in the supernatant fraction was determined using the Bradford Reagent (Carl Roth, Karlsruhe, Germany). For solubility studies 70 μg of Tris-soluble protein extracts were incubated with 1 μl PK (Ambion, Thermo Fisher Scientific), diluted to either 1:10,000 or 1:30,000 in PBS, resulting in final activity of 1.2 or 0.4 × 10^-6^ U PK per μg total brain protein. Digestion was carried out for 15 min at 37°C and reaction stopped by cooling down on ice. Protein extracts were separated in 10% Tris-tricine SDS-PAGE, transferred to PVDF membrane by semi-dry blotting at 4°C, and membranes incubated with primary mAb 204, mAb D37A6 and mAb 3H2897 followed by appropriate secondary antibody (Dako, Glostrup, Denmark). Chemiluminescent detection was carried out according to the manufacturer’s instructions (GE Healthcare, Little Chalfont, United Kingdom). Brain tissue extracts were analyzed in a different SDS-PAGE system to that used for N1E-115 cells. The major α-Syn band present in mouse brain and N1E-115 cells showed identical mobility when separated by either of the gel systems (not shown). The 19-kDa α-Syn band, however, migrated with an apparently lower molecular weight (15 kDa) when separated using the Tris-tricine SDS PAGE system described in this section.

### Immunohistochemistry and Stereological Cell Counting of Brain Sections

Procedures for staining α-Syn in brain sections were conducted as described previously (Melis et al., 2015a,b). Brain hemispheres were coronally sectioned (5 μm) at desired levels. For each animal, three sections were examined and to avoid re-counting of the same cell, every fourth section was counted. Sections were boiled in citric acid-sodium citrate buffer (10 mM; pH 6.0), transferred to peroxidase-blocking solution before mAb 204 and secondary biotinylated antibodies were applied. Thiazin red (0.001%) was included with primary antibody as an indicator of aggregation state of α-Syn (Mena et al., 1995, 1996). Sections were developed in diaminobenzidine solution (Dako) and counter stained with Ehrlich haematoxylin solution (Carl Roth). Sections of individual brains were stained simultaneously and viewed by light microscopy using a Leica DM LB2 or a Zeiss Axiovert 135 microscope at 50x- to 200x- magnification. α-Syn-positive cells were counted by an investigator blind to the experimental details for three sections per animal in the following regions of interest (Franklin and Paxinos, 2008): Hip+S, auditory and visual cortices, SNpR, RMC, InG and DpMe+MGV at Bregma -3.8 mm; MC at Bregma +0.74 mm; and SMC at Bregma -0.70 mm. Cohort sizes for each drug condition are given in **Table 2**.

For PK-digestion studies, sections of cortex on slides were incubated for 15 min with PK (diluted in PBS to give a final activity of 1.7 × 10^-4^ and 3.4 × 10^-4^ U per slide) at 37°C and immunodetection with mAb 204 was conducted thereafter as described above. For thiazin red staining, cortical sections were dewaxed, incubated with mAb 204, followed by incubation in appropriate fluorochrome-conjugated secondary antibody. Thiazin red solution (0.001%) was applied for 15 min at room temperature and slides mounted with Gold Antifade Reagent (Cell Signaling).

### Measurement of MT^+^ Levels in the Brain

Total MT^+^ level was measured in brain samples by means of a modified DCE liquid-liquid extraction method (modified from Peter et al., 2000). Briefly, half brains were homogenized in 3x the weight of water to brain. To a 200-μl sample of brain homogenate, 250 μl 0.45 M orthophosphoric acid was added and heated for 30 min at 70°C; Sodium hexane sulfonate (100 μL; 5% w/v in water) and 0.5 ml DCE were added and the mixture incubated for 30 min at room temperature. The mixture was separated by centrifugation and a 300-μl sample of the DCE layer was dried down and the residue suspended in mobile phase (50:50 water:acetonitrile containing 0.05% formic acid). Total MT^+^ levels were determined by UPLC with LC-MS/MS detection. The lower limit of detection was 1 ng/g.

### Behavioral Testing

Animal behavior was assessed in the light/dark box. A gray colored Perspex box (43 cm × 26 cm × 26cm: L × W × H) was divided into two equal-sized areas separated by a partitioning wall containing an aperture to enable free access to either side. One compartment was covered by a lid (dark zone) while the second one remained open (light zone). To create a stressful situation, mice were i.p. injected with saline (0.9%) and immediately released in one corner of the light compartment and allowed to explore both compartments freely for 5 min. Ambulation within the light compartment was recorded by an overhead camera and analyzed using EthoVision XT10 (Noldus IT, Wageningen, Netherlands). Dependent variables included time spent in each compartment (sec), velocity of movement in the light box (cm/s), cumulative time of immobility (%) and meander as a measure of path directionality (degree/cm). Behavioral assessment took place during the last week of drug dosing. The light/dark box was cleaned using 70% ethanol between subjects. For cohort sizes, see **Table 3**.

### Data Analyses

Analysis for stereological cell counting was performed using R software environment (cell counts; R Development Core Team, 2004), with linear models focussing on the variation between vehicle group and normalization of output by drug treatment. The natural logarithm of (cell count+1) was modeled, since it is more normally distributed. Analyses in **Figures 6, 7** were also performed using R software, again using linear modeling. All other statistical analyses were conducted using GraphPad Prism (version 6.00; GraphPad Software Inc., United States). Data are presented as mean ± SE. Data were analyzed by Analysis of Variance (ANOVA) and Bonferroni corrected *t*-test. Differences were considered to be statistically significant at *p* < 0.05.

## Results

### Aggregated α-Syn Accumulates in Differentiated N1E-115 Cells Expressing h-α-Syn

**FIGURE 1:**
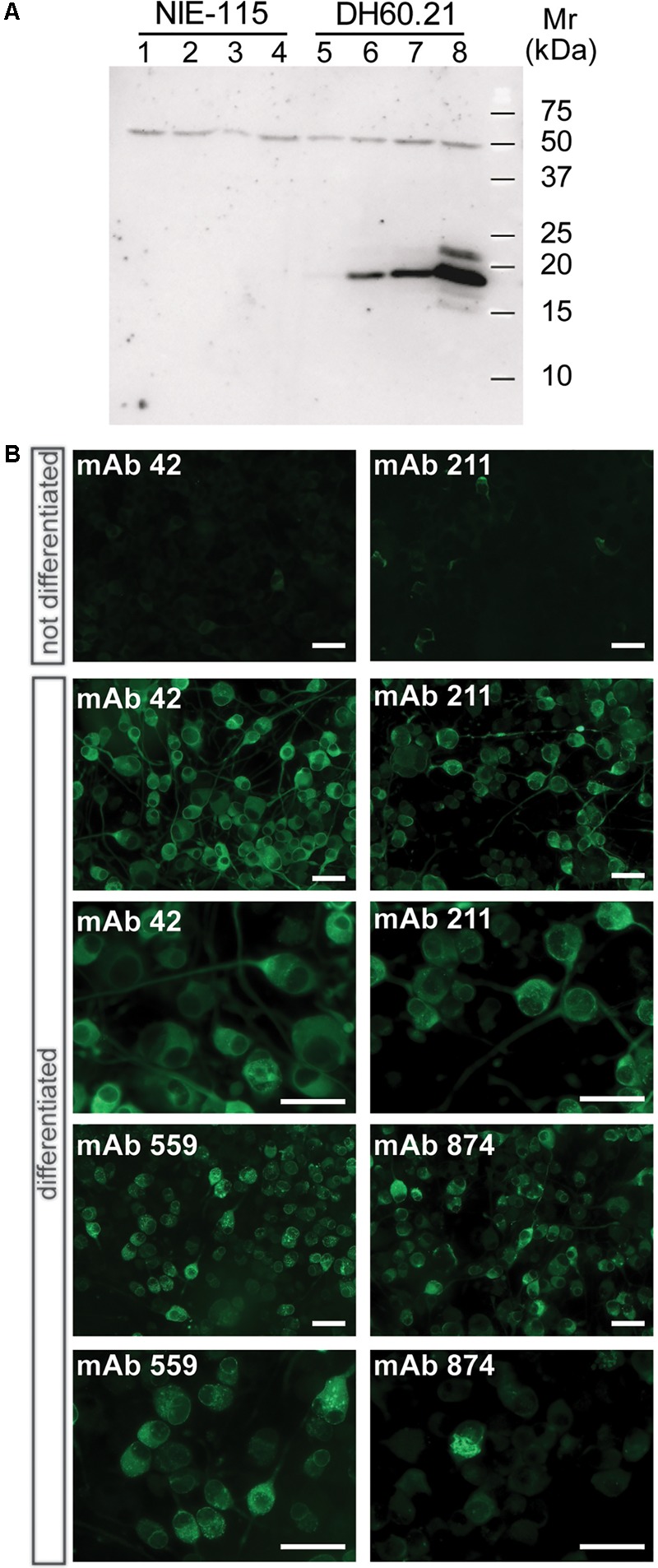
α-Syn expression in differentiated neuroblastoma cells as granular and aggregated α-Syn. **(A)** N1E-115 cells were transfected with the constitutive SSFsyn construct containing full-length h-α-Syn fused to a membrane-targeting signal sequence. Samples were analyzed by SDS-PAGE and immunodetection of α-Syn using mAb 42. Lanes 1-4, non-transfected N1E-115 cells; lanes 5-8, DH60.21 cells with SSFsyn. Lanes 1 and 5, undifferentiated cells grown in the presence of 10% serum; lanes 2 and 6, cells differentiated by depletion of serum (1%); lanes 3 and 7, cells differentiated in the presence of 1% serum plus 100 ng/mL NGF; lanes 4 and 8, cells differentiated in medium containing 1% serum plus 1mM db-cAMP. Non-transfected N1E-115 cells showed no expression of α-Syn even after differentiation (lanes 1–4). In contrast, transfected DH60.21 cells showed low levels of α-Syn expression that increased following differentiation (lanes 6–8), and the greatest levels were observed following differentiation using db-cAMP as indicated. **(B)** Immunocytochemistry of DH60.21 cells before and after differentiation in the presence of 1% serum plus 1mM db-cAMP. Cells, fixed with 3% paraformaldehyde, were permeabilised with Triton X-100 and shown to be labeled by four α-Syn mAbs, as indicated. Images are shown at two levels of magnification to first demonstrate extent of staining of cells and then, secondly, to provide detail of granular or fibrillar staining within specific cells. The epitopes recognized by the mAbs are provided in **Table 1**.

When lysates of N1E-115 neuroblastoma cells were separated by Tris-glycine SDS-PAGE, no α-Syn was detected using mAb 42 in immunoblots regardless of whether or not cells had been differentiated (**Figure 1A**, lanes 1–4). Likewise, the level of immunoreactivity was minimal in the DH60.21 cell line, derived from N1E-115 mouse neuroblastoma cells and constitutively expressing full-length human α-Syn fused with an N-terminal signal sequence peptide (SSFsyn), in the absence of differentiation (**Figure 1A**, lane 5). A mAb 42-reactive band, having a relative mobility of 19-kDa consistent with h-α-Syn (Jakes et al., 1994), was detected following differentiation using either serum depletion alone (**Figure 1A**, lane 6), or serum depletion plus either 100 ng/ml NGF (**Figure 1A**, lane 7), or 1 mM db-cAMP (**Figure 1A**, lane 8); the greatest levels were obtained following differentiation in medium containing 1 mM db-cAMP and 1% serum. A 50-kDa band was also labeled using mAb 42. This originates from non-specific antibody binding since no α-Syn sequence was obtained in this area by mass spectrometry. No high molecular weight aggregates were observed in lysates, using SDS-PAGE.

The above findings were consistent with immunocytochemical studies. Undifferentiated D60.21 cells showed little evidence of labeling of endogenous α-Syn using several different anti-α-Syn antibodies (shown for two mAbs in **Figure 1B**, upper panel) and the levels were indistinguishable from cells in which primary antibody had been omitted (data not shown). α-Syn immunolabelling was substantially increased following differentiation of D60.21 cells (**Figure 1B**). The pattern of immunoreactivity indicated the presence of both diffuse, cytoplasmic labeling as well as granular labeling around the nucleus and in the region of the axon hillock (**Figure 1B**).

### LMTM Inhibits h-α-Syn Aggregation in Differentiated DH60.21 Cells

**FIGURE 2:**
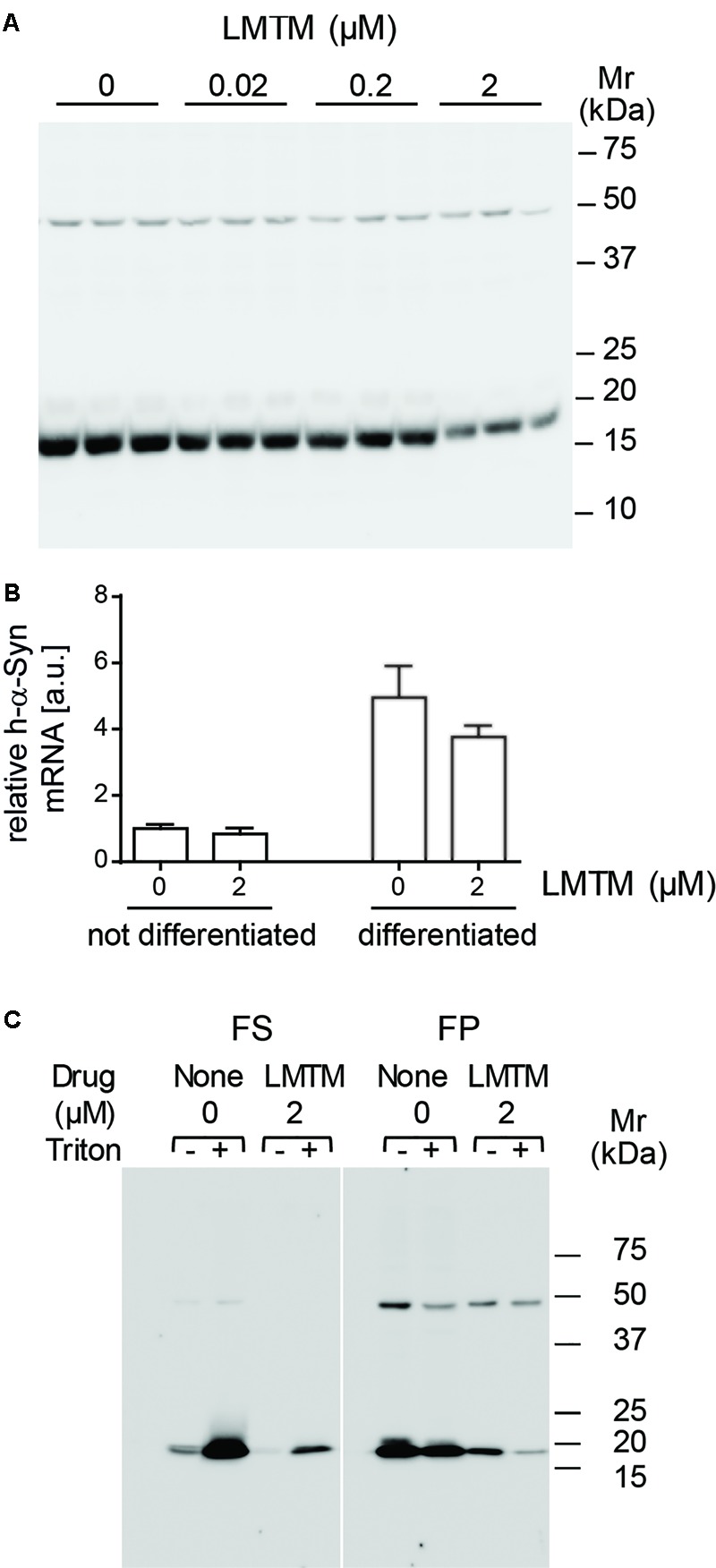
LMTM treatment decreased expression and altered solubility of the h-α-Syn. **(A)** DH60.21 cells were differentiated in medium containing 1% serum and 1 mM db-cAMP in the presence of LMTM, as indicated, for 3 days in triplicate. The amount of h-α-Syn on immunoblots decreased with increasing concentrations of LMTM. **(B)** Cells treated with or without LMTM (2 μM) were prepared similarly for detection of α-Syn mRNA. RNA was extracted from frozen cell pellets by TRIzol and quantified by Maxima SYBR Green. The relative level of α-Syn m-RNA was normalized to GAPDH and values are expressed as mean ± SE. **(C)** For the analysis of protein solubility, differentiated DH60.21 cells were treated in the presence or absence of 2 μM LMTM for 3 days. α-Syn was extracted in Tris buffer in the absence or presence of 0.1% Triton X-100 and cell debris sedimented by low-speed centrifugation. The resultant supernatant was subjected to centrifugation at 353,000 × *g* for 60 min to provide fast supernatant (FS) and fast pellet (FP) fractions. Samples for **(A,C)** were analyzed using Tris-glycine SDS-PAGE and immunodetection of α-Syn using mAb 42.

The level of the 19-kDa h-α-Syn species relative to the non-specific 50-kDa immunoreactivity was reduced in differentiated DH60.21 neurons following treatment with LMTM. A representative immunoblot is shown in **Figure 2A**. The EC_50_ value (±SE) for LMTM was 1.11 ± 0.28 μM (*n* = 6). As shown in **Figure 2B**, LMTM had either no or minimal effect on expression of h-α-Syn mRNA in undifferentiated and differentiated neurons, respectively. Baicalein, another inhibitor of α-Syn aggregation in a cell-free model (Zhu et al., 2004; Masuda et al., 2006), was also tested in differentiated DH60.21 cells. This also decreased the level of 19-kDa immunoreactivity retained on the immunoblot, and the corresponding EC_50_ value (3.13 ± 1.37 μM [*n* = 2]) was approximately threefold greater than with LMTM. Less favorable than baicalein was the efficacy of the triphenylmethanes Coomassie Brilliant Blue G and R. In the thioflavin T assay, the EC_50_ values were 5.4 and 322 μM for the G and R dyes, respectively (Ahsan et al., 2017).

The state of h-α-Syn expressed in cells was partially characterized by differential centrifugation and detergent extraction using Triton X-100 in the presence or absence of LMTM treatment (**Figure 2C**). In the absence of Triton X-100, the majority of h-α-Syn sedimented with low-speed centrifugation. Cellular membranes and the h-α-Syn in the low-speed supernatant was largely (80%) sedimentable following high-speed centrifugation at 353,000 x g for 60 min (11.7 Svedberg units, FP fraction in **Figure 2C**) consistent with an implied molecular weight larger than 200 kDa. When Triton X-100 was included in the extraction, the majority of h-α-Syn was released into the low-speed supernatant, of which a substantial portion (36%) remained in an aggregated state sedimenting in the FP fraction. Following treatment with LMTM in the absence of Triton X-100, the overall quantity of h-α-Syn was reduced by approximately 85% and this was found exclusively in the FP fraction following high-speed centrifugation. When LMTM treatment was combined with Triton X-100, almost all of the remaining h-α-Syn was transferred to the high-speed supernatant (FS) fraction. Quantitative densitometry showed that levels of the non-specific 50-kDa band were not affected by the addition of LMTM (data not shown).

### LMTM Decreases the Numbers of α-Syn-Positive Cells in Multiple Brain Regions

**FIGURE 3:**
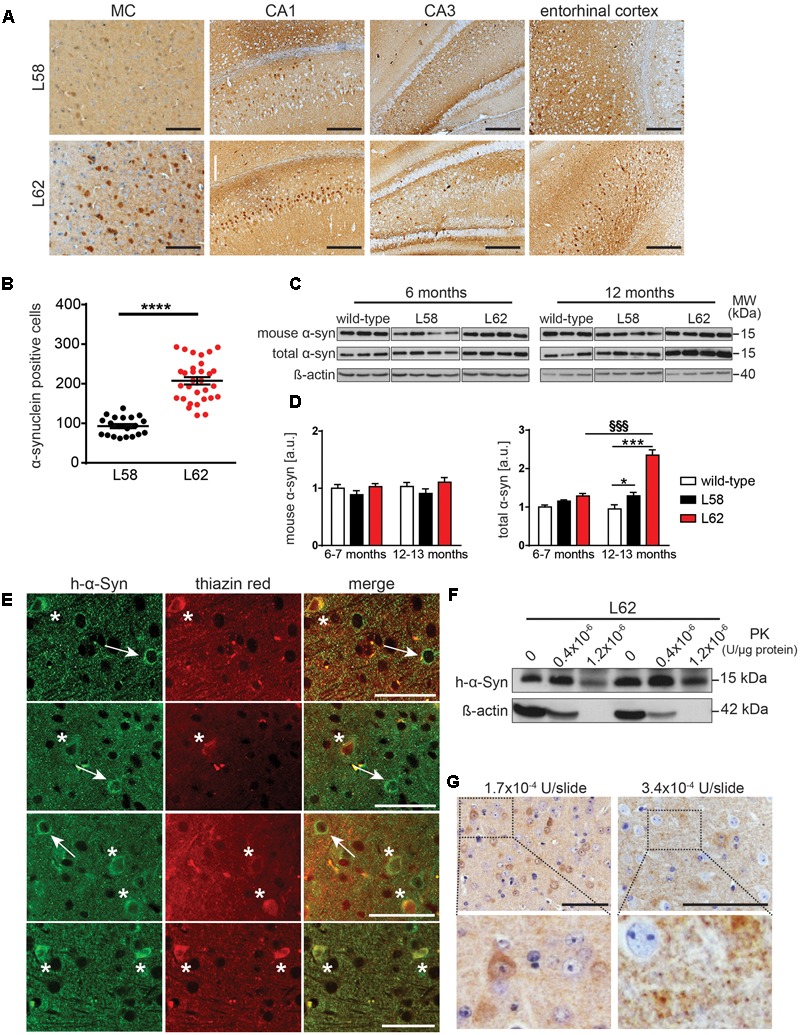
α-Syn expression in transgenic mice increased with age and was greatest in L62 mice. **(A)** Expression of h-α-Syn in L62 was greater than in L58. Representative microphotographs taken from primary motor cortex, hippocampal CA1/CA3 and entorhinal cortex revealed differential α-Syn immunoreactivity (mAb 204) between transgenic lines aged 6 months. **(B)** Quantification of α-Syn positive cells (mean ± SE) in the right hemisphere of the midbrain using mAb 204, showing more than twofold greater number of cells with h-α-Syn inclusions in L62 than in L58 mice aged 6 months (unpaired Student *t*-test: ^∗∗∗^*p* < 0.0001). **(C)** Assessment of total and murine α-Syn (normalized to β-actin loading) in L58 and L62 mice using immunoblots revealed unchanged murine α-Syn levels in either line but a progressive increase in total α-Syn in both lines between 6 and 12 months due to the build-up of transgenic human α-Syn, with a greater accumulation of α-Syn in L62 than in L58, as shown by densitometric quantification of α-Syn normalised to β-actin in **(D)**. Values are expressed as mean ± SE. Bonferroni post-test: ^∗^*p* < 0.05 and ^∗∗∗^*p <* 0.001 for genotype effect and §§§, *p* < 0.001 for age effect. **(E)** Representative images for L62 motor cortex sections co-stained with anti-α-Syn mAb 204 (left) and thiazin red (middle), showing a neuron positive for α-Syn (arrow) and another neuron in which α-Syn inclusions were co-labelled with thiazin red (asterisk). **(F)** Tris-soluble monomeric α-Syn was resistant to PK cleavage, whereas β-actin was not. **(G)** Representative images for L62 motor cortex sections stained with mAb 204 treated with PK prior to antibody reaction show cytoplasmic and synaptic α-Syn inclusions resistant to PK cleavage. Samples for **(C,F)** were analyzed using Tris-tricine SDS-PAGE and immunodetection of α-Syn using mAb 204 (h-α-Syn), mAb D37A6 (mouse α-Syn) and mAb 3H2897 (total α-Syn). Scale bars, 100 μm.

In both L58 and L62 mice expressing full-length α-Syn fused with a signal peptide, α-Syn immunoreactivity was found predominantly in pyramidal neurones of the neocortex and hippocampus, as well as in the principal neurones of the substantia nigra, the red nucleus, superior colliculus and geniculate nuclei (**Figure 3A**). Wild-type mice did not show any immunoreactivity with any of the antibodies tested (Frahm et al., 2017). α-Syn immunoreactivity in L62 was more widespread and affected significantly more cells than in L58 (*p* < 0.0001, **Figure 3B**). In addition to the intraneuronal staining, α-Syn immunoreactivity was also present throughout the neuropil of transgenic mice, consistent with its abundance in synapses. Staining of wild-type mice revealed no such synaptic staining with mAb 204 (see below).

Levels of endogenous murine α-Syn and transgenic h-α-Syn were examined by immunoblotting in a urea-soluble brain extract after low-speed centrifugation using mAbs D37A6 and mAb 3H2897 to label murine and total α-Syn, respectively. Overall, both genotype and age-effects were significant (*p* < 0.001). There was no increase in murine α-Syn levels in either L58 or L62 relative to wild-type controls at 6–7 months or 12–13 months. However, total brain α-Syn was significantly increased in older L62 mice (*p* < 0.001), implying that the age-dependent increase was due to accumulation of the transgenic h-α-Syn in brain (**Figures 3C,D**). In L58 mice, age-related accumulation of total α-Syn was less pronounced and not statistically significant, although distinct from wild-type mice at 12–13 months (**Figure 3D**, *p* < 0.05).

Immunohistochemistry using mAb 204 showed diffuse granular and perinuclear labeling, as well as more homogeneous labeling in the cell body of some neurones. The latter were dual labeled with thiazin red, implying transition to fibrillar aggregates (Mena et al., 1995, 1996). (**Figure 3E**). In order to confirm whether the aggregation detected histologically was associated with increased resistance to proteases, Tris-soluble protein lysates were treated with PK. A large amount of Tris-soluble h-α-Syn remained intact while ß-actin was completely cleaved (**Figure 3F**). Following similar treatment of histological sections using higher concentrations of PK, both the diffuse granular and somatic α-Syn remained intact (**Figure 3G**).

**FIGURE 4:**
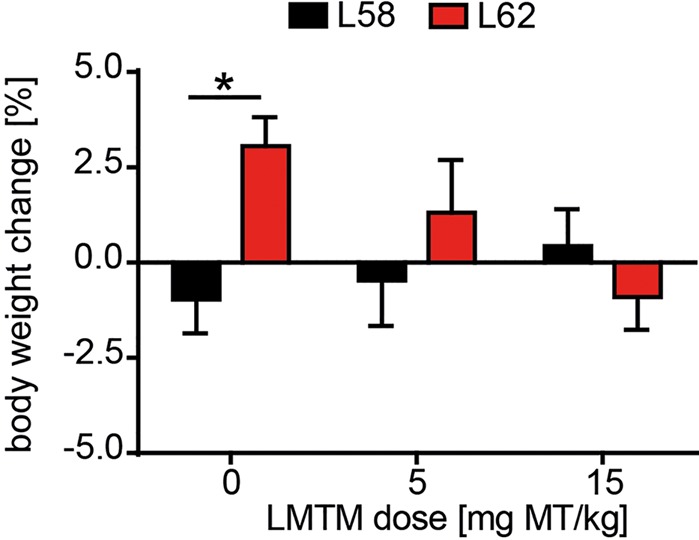
LMTM was tolerated by mice. Effect of LMTM on body weight for L58 and L62 mice is shown. Body weight gain/loss is expressed as the mean percentage change (±SE) over 6 weeks relative to the start of treatment. Drug exposure did not differ from vehicle for either genotype but there was a significant difference (*p* = 0.04) in body weight gain between vehicle-treated mice for both lines.

Having established a progressive histopathological phenotype in both L58 and L62 mice, we examined the effect of LMTM in these mice after oral treatment at 5 and 15 mg MT/kg/day for 6 weeks. No adverse effects were observed at either dose, although L62 mice had reduced body weight gain at the higher dose (**Figure 4**). The mean brain levels of MT^+^ were 30.62 ± 27.34 ng/g, and 100.49 ± 95.16 ng/g for the 5 and 15 mg MT/kg doses, respectively.

**FIGURE 5:**
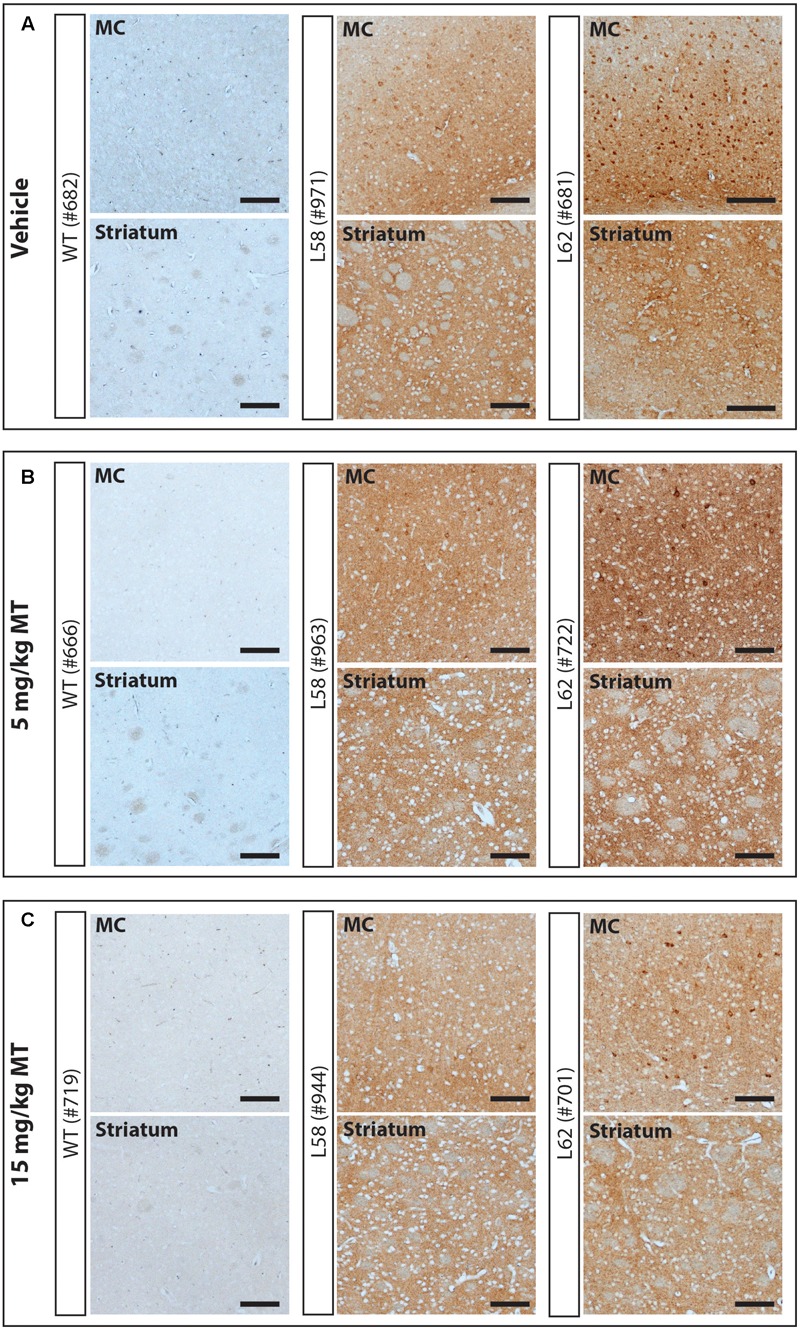
LMTM decreased α-Syn pathology in L58 and L62 mice. Representative immunohistochemical images of the motor cortex (MC) and striatum for vehicle- **(A)** and LMTM-treated **(B,C)** mice. WT mice were devoid of synuclein immunoreactivity. L58 and L62 showed both neuronal α-Syn inclusions and extensive synaptic content; that were both decreased with increasing dose in these representative blots. Scale bar, 100 μm.

**FIGURE 6:**
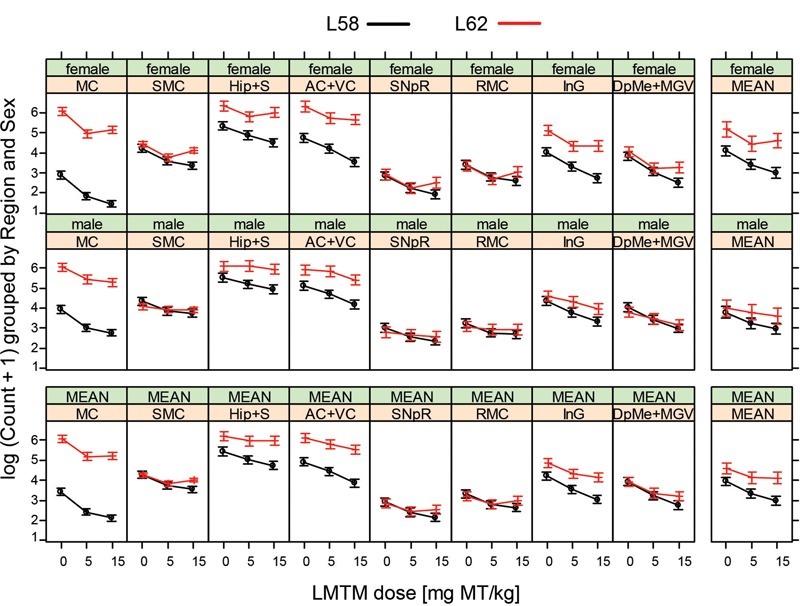
LMTM decreased α-Syn pathology in L58 and L62 mice in multiple brain regions. LMTM significantly lowered the number of mAb 204-immunoreactive α-Syn-positive cells in multiple brain regions in L58 and L62 mice of both sexes. Values are expressed as mean log(count +1) (±SE).

WT mice had no α-Syn-reactive cells nor any synaptic staining (**Figure 5**) and the number of α-Syn-reactive cells and the level of synaptic staining in L58 and L62 mice were qualitatively decreased after LMTM treatment (**Figure 5**). The number of α-Syn-reactive cells was decreased significantly after treatment with LMTM at both doses, and the extent of reduction was comparable for both cortical and sub-cortical regions (**Figure 6** and **Table 4**). The decrease was greater in L58 than in L62 and, in L62, was greater in females than in males. Expressed as log(cell count + 1), the effect sizes at each dose for L58 were -0.61 and -0.96 and for L62 were -0.47 and -0.48 for doses of 5 and 15 mg MT/kg, respectively). These represent reductions in the range of 38–63%, when L58 and L62 are both considered, relative to vehicle-treated controls.

**Table 4:** Effect size for LMTM on α-Syn pathology^∗^.

Genotype	LMTM dose (mg MT/kg)	Sex	Effect size	*SE*	*p*-value
L58	5	F	-0.67521	0.081539	2.443e-16^∗∗∗^
L58	15	F	-1.08321	0.078948	<2.2e-16^∗∗∗^
L58	5	M	-0.54608	0.092228	3.861e-09^∗∗∗^
L58	15	M	-0.82816	0.098710	<2.2e-16^∗∗∗^
L58	5	Mean	-0.61064	0.059163	<2.2e-16^∗∗∗^
L58	15	Mean	-0.95569	0.058661	<2.2e-16^∗∗∗^
L62	5	F	-0.72698	0.079024	<2.2e-16^∗∗∗^
L62	15	F	-0.56317	0.083936	2.651e-11^∗∗∗^
L62	5	M	-0.21098	0.086082	0.01435^∗^
L62	15	M	-0.39943	0.081572	1.067e-06^∗∗∗^
L62	5	Mean	-0.46898	0.055217	<2.2e-16^∗∗∗^
L62	15	Mean	-0.48130	0.054266	<2.2e-16^∗∗∗^

*^∗^Effect size, expressed as log(count+1), shown for means over all regions in individual transgenic lines (mean effect in male and female) and in individual genders. ^∗^*p* < 0.05; ^∗∗∗^*p* < 0.001.*

### LMTM Targets Anxiety and Movement-Related Aspects in a Synucleinopathy Mouse Model

**FIGURE 7:**
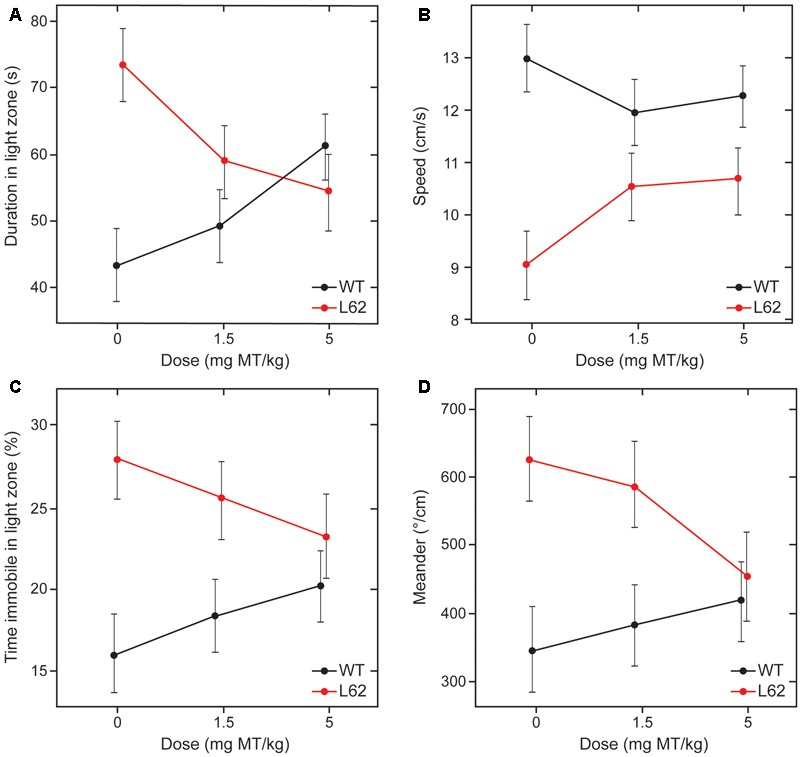
LMTM rescued behavioral deficiencies seen in L62 mice during the light/dark box testing. L62 mice expressed an anxiolytic phenotype as measured by four parameters: **(A)** Time spent in the illuminated compartment of the light/dark box; **(B)** speed of movement; **(C)** immobility; and **(D)** meander as a stereotypic trait. The phenotype observed in L62 mice was attenuated with LMTM at doses of ≥1.5 mg MT/kg, with the exception of meander, where the difference remained significant at the 1.5 mg MT/kg dose. However, WT controls also were affected by the administration of MT, but this was only significant for the proxy ‘duration in light zone’. For details, see Results.

Behavioral effects of LMTM treatment were examined in L62 only. Transgenic mice and wild-type controls were assessed behaviorally in the light/dark box following oral treatment with LMTM (1.5 and 5 mg MT/kg) for 6 weeks. Compared to wild-type controls, vehicle-treated L62 mice spent more time in the illuminated compartment of the light/dark box (maximum time 300 s; **Figure 7A**; ^∗∗∗^*p* < 0.001), had reduced speed of movement (**Figure 7B**; ^∗∗∗^*p* < 0.0001), were immobile longer in the light zone (**Figure 7C**; ^∗∗∗^*p* < 0.001) and showed greater stereotypic meandering (**Figure 7D**; ^∗∗^*p* < 0.01). WT control mice showed a dose-dependent increase in time spent in the light zone (^∗^*p* < 0.05; dose-dependent), but effects on the other parameters tested were not significant. L62, on the other hand, showed a significant dose-dependent reduction in time spent in the light zone (**Figure 7A**; ^∗^*p* < 0.05). The treatment effects on other parameters were in the direction of normalization behavior, particularly for the 5 mg MT/kg dose.

**FIGURE 8:**
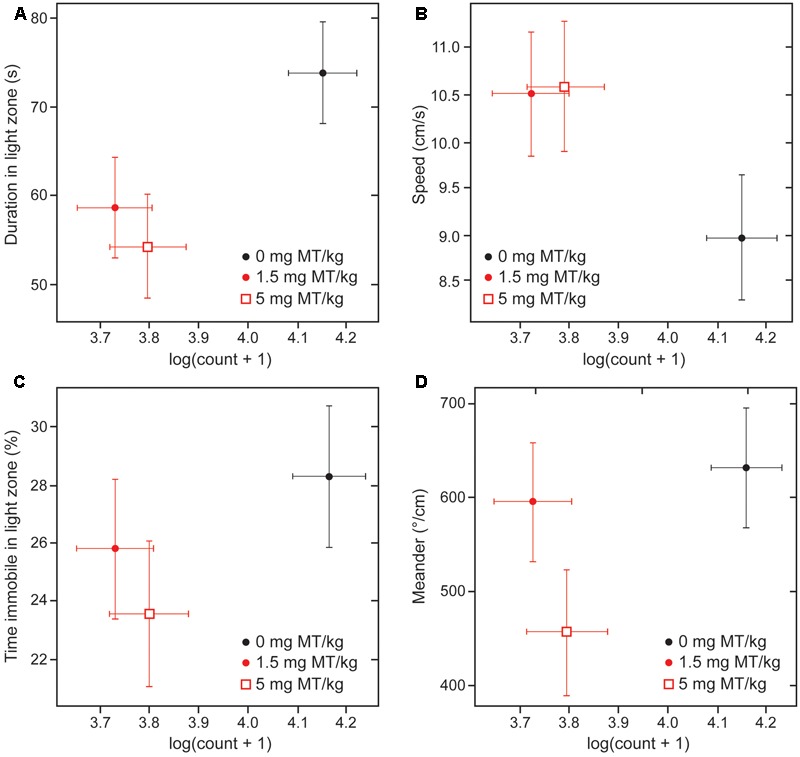
Behavioral rescue with LMTM is concomitant with decrease in immunoreactive α-Syn inclusions. The four behavioral parameters measured in the light/dark box are compared as a function of mAb 204-reactive α-Syn pathology in motor cortex of L62 mice: **(A)** duration in light zone; **(B)** speed; **(C)** time immobile in light zone and **(D)** meander. There was a significant decrease in numbers of α-Syn-positive cells at both doses (^∗∗∗^*p* < 0.0001 for 1.5 mg MT/kg; ^∗∗^*p* < 0.01 for 5 mg MT/kg). The decrease in α-Syn-reactive cells was associated with an improvement of the anxiolytic measures. The effect observed with 5 mg MT/kg did not exceed that observed with 1.5 mg MT/kg, with the exception of the parameter ‘meander’ **(D)**.

There was an association between the beneficial effects of treatment on behavior and the decrease in numbers of α-Syn-positive cells at both active doses (**Figure 8**; ^∗∗∗^*p* < 0.0001 for 1.5 mg MT/kg and ^∗∗^*p* < 0.01 for 5 mg MT/kg, with respective effect sizes of -0.42 and -0.35 in units of log(cell count + 1)). The effects of LMTM were generally comparable at the two doses, both in terms of behavioral rescue and reduction in α-Syn pathology.

## Discussion

We report the development of two novel model systems for testing the efficacy of synuclein aggregation inhibitors as a potential treatment for diseases characterized by pathological aggregation of synuclein, particularly PD and dementia with Lewy bodies. In these diseases, synuclein pathology spreads from brainstem, via the midbrain to neocortex, with development of symptom profiles corresponding to the spread of pathology (Braak and del Tredici, 2016). The formation of pathological aggregates after initial seeding or nucleation, and subsequent prion-like spread of pathology is emerging as a common theme for a group of progressive neurodegenerative protein misfolding disorders which also include Alzheimer’s disease and Huntington’s chorea (Lee S.J. et al., 2010). Inhibition of pathological aggregation provides a rational therapeutic approach for these diseases, with a view to limiting both the direct toxic effects of aggregates and their prion-like spread. We have previously reported that the methylthioninium moiety (MT) acts as an inhibitor of tau protein aggregation in cell-free and cell-based models *in vitro* (Harrington et al., 2015). We have also shown that MT reduces tau aggregation pathology and normalizes behavioral deficits in tau transgenic mouse models (Melis et al., 2015a). These studies formed the basis for clinical trials in AD first using the oxidized form of MT (present in methylthioninium chloride, MTC, commonly known as methylene blue) (Wischik et al., 2015), and then the stable reduced form (LMTM). The latter was developed to overcome the absorption limitation of MTC, and was found to have potential efficacy on clinical and brain imaging endpoints as monotherapy even at a dose as low as 4 mg twice a day (Gauthier et al., 2016; Wilcock et al., 2018).

Cell-based models of aggregation provide an important intermediate testing platform which permit determination of activity and toxicity within the milieu of the living cell. We previously reported that pathological aggregation and truncation of tau protein could be induced using a fusion construct incorporating a signal sequence which directs the protein to the endoplasmic reticulum (Harrington et al., 2015). This redirection is considered to enhance the seeding of aggregation by providing an initial abnormal binding substrate (Lai et al., 2016). Because of the inherent toxicity of the aggregates produced, generation of stable cell lines requires a combination of low level constitutive expression of the protein and a further intervention which increases expression levels and aggregation in a controlled manner which permits drug testing. In the case of α-Syn, we found that it is possible to express an aggregation-prone construct constitutively at low levels in the N1E-115 mouse neuroblastoma cell line, and to induce aggregation following differentiation. There have been similar findings reported for SH-SY5Y neuroblastoma cells overexpressing α-Syn, where differentiation was required to generate inclusion bodies (Hasegawa et al., 2004). In the NIE-derived model, differentiation is associated with a substantial increase in the level of expression of h-α-Syn mRNA and a corresponding increase in the level of h-α-Syn protein. Labeling with anti-α-Syn antibodies showed the presence of granular synuclein aggregates in a perinuclear location, in the axon hillock and diffusely throughout the cytoplasm following differentiation. The sedimentation characteristics of the aggregates were consistent with the formation of decamers or greater oligomers with a molecular weight greater than 200 kDa.

Treatment with LMTM decreased the overall quantity of SDS-soluble α-Syn retained on immunoblots with an EC_50_ of about 1 μM, and largely eliminated the high molecular weight oligomers from the high-speed pellet. Baicalein, previously reported to have anti-aggregation properties for synuclein (Zhu et al., 2004; Hu et al., 2016), produced similar effects in this cell model, except with a higher EC_50_ of about 3 μM. We have also previously reported similar results for LMTM using signal sequence fusion constructs of tau protein (Harrington et al., 2015). Therefore, the fusion protein construct provides a useful means of inducing α-Syn aggregation at the cellular level, and this aggregation can be blocked by LMTM at concentrations consistent with dosing *in vivo*.

Based on the cell model findings, we developed two corresponding transgenic mouse models, L58 and L62. L62 had higher brain levels of α-Syn and more widespread pathology. The level of α-Syn found in the brain also increased in an age-dependent manner. Using differential labeling for endogenous mouse and total α-Syn, we found that the age-dependent increase was due to selective accumulation of the human form in L62. The aggregates were found predominantly in the pyramidal cells of the cortex, and in the principal cells of a number of subcortical nuclei. The immunohistochemical features of the aggregates were similar to those produced in the NIE-derived cell line, with granular perinuclear aggregates and more diffuse cytoplasmic labeling which could be double labeled with thiazin red with sufficiently high levels of aggregation suggesting a transition to fibrillar forms (Mena et al., 1995, 1996). We further confirmed that the aggregates produced in the brain were more resistant to PK than actin, a finding previously reported in other transgenic synuclein mouse models.

As found in the cell model, treatment with LMTM resulted in an overall decrease in levels of α-Syn aggregation pathology, in the range of 38–63% compared with vehicle-treated animals. The effect of treatment was greater in the less severely affected L58 and, in L62, the effect was greater in females. These beneficial effects on pathology were associated with normalization of the abnormal behavioral phenotype seen in L62 mice which is described more fully in a separate report (Frahm et al., 2017). L62 mice are characterized by general poverty of movement, seen as longer time spent in the light zone of a light/dark box, longer periods of immobility in the light zone, reduced speed of movement, and greater stereotypic meandering movement, all of which were to varying extents reversed by treatment with LMTM. Direct comparison of the effects of LMTM at 1.5 and 5 mg/day again confirmed a lack of dose response on behavior, and an association between effects on pathology and behavior compared with vehicle-treated control mice.

Although LMTM dosing at 5 and 15 mg/day produced a threefold difference in brain levels of MT, there was no evidence of a dose-response for effect on pathology. There appears therefore to be an overall lack of dose-response over a 10-fold dosing range from 1.5 to 15 mg MT/kg. This lack of dose-response was also found for tau pathology in tau transgenic mouse models using LMTM, where there was even evidence of a negative dose-response at 45 mg MT/kg (Melis et al., 2015a). Indeed, the same lack of dose-response was also found in a recent clinical trial over a 30-fold dosing range of 8 mg/day to 250 mg/day. This suggests that, at least in the brain, the effect of treatment depends on reaching a critical threshold concentration of MT beyond which there is no advantage to using higher doses. It is possible from this study to estimate this threshold as approximately 15 ng MT/g (0.05 μM MT). Interestingly, this is also the concentration required for enhancement of autophagy (Congdon et al., 2012) and of oxidative phosphorylation by mitochondria (Zhang et al., 2006), although much lower than the EC_50_ found in the NIE cell line. This suggests that the cell model, although useful for predicting efficacy *in vivo*, may over-estimate the concentration required for activity in the much more complex milieu of the whole brain.

α-Syn has been expressed in various forms in transgenic mice. There are transgenic mice expressing full-length α-Syn containing those point mutations (A53T, A30P or E46K) (Sommer et al., 2000; Rockenstein et al., 2002; Piltonen et al., 2013; Deusser et al., 2015; Mbefo et al., 2015) found in dominant familial PD (Dauer and Przedborski, 2003). Expression of wild-type h-α-Syn in mice has been achieved under the regulatory control of either the platelet-derived growth factor B or *Thy1*-promotors (Masliah et al., 2000; van der Putten et al., 2000). The loss of dopaminergic cells and presence of α-Syn inclusions in neurons was directly correlated with the level of h-α-Syn transgene expression (Masliah et al., 2000; van der Putten et al., 2000). Although the α-Syn transgene in this study was expressed in various brain regions relevant to parkinsonian phenotypes, there was no frank cell loss in the substantia nigra (Frahm et al., 2017). This is consistent with the finding that overexpression of C-terminally truncated α-Syn (residues 1–120) in mice leads to pathological changes in the nigrostriatal projections, without any associated cell loss in the pars compacta by 12 months (Tofaris et al., 2006). Moreover, global expression of protein throughout the brain is a feature of the *Thy1*-promotor when used as a regulatory element to drive the α-Syn transgene expression (Kahle et al., 2002; Rockenstein et al., 2002; Magen et al., 2012; Delenclos et al., 2014) and L58 and L62 mice in this study expressed α-Syn in brain stem, cortical and subcortical structures.

Mouse models of wild-type and mutant α-Syn presenting with neurodegeneration have been proposed as suitable for the development and assessment of disease-modifying therapies for idiopathic PD and dementia with Lewy bodies (Allain et al., 2008). There has been particular interest to intervene with the misfolding and oligomerisation α-Syn using antibodies to reduce the α-Syn burden in transgenic mice (Bae et al., 2012; Lindström et al., 2014; Mandler et al., 2014; Tran et al., 2014) and to prevent the cell-to-cell transmission of α-Syn between neurons (Desplats et al., 2009; Danzer et al., 2012; Luk et al., 2012). Alternative strategies include the drug-induced increase of α-Syn clearance through enhancement of proteasomal or lysosomal activation (Lee B.H. et al., 2010), or by stimulation of autophagy (Dehay et al., 2015). Targeting α-Syn disaggregation (Sivanesam et al., 2015) is not without risk given the fact that oligomeric proteins are considered neurotoxic and fibril formation may be a safe way for disposal of oligomers. Nevertheless, there are several compounds that inhibit α-Syn aggregation *in vitro* (Zhu et al., 2004; Masuda et al., 2006; Wagner et al., 2013; Ibrahim and McLaurin, 2016) and which demonstrate activity *in vivo* using mouse models of PD (Hu et al., 2016; Tatenhorst et al., 2016). There was no evidence of increased general toxicity or neurotoxicity for LMTM over a 10-fold dosing range up to 15 mg MT/kg.

The combination of beneficial effects on pathological aggregation of α-Syn, enhancement of autophagy (Congdon et al., 2012) and enhancement of mitochondrial respiration makes LMTM a particularly interesting candidate for further clinical study in both PD and dementia with Lewy bodies. The toxicology of LMTM has been well characterized in preclinical studies, and a relatively benign safety profile of LMTM has been well documented now in Phase 2 and Phase 3 trials (Wischik et al., 2015; Gauthier et al., 2016; Wilcock et al., 2018). The animal safety data available from the present study adds to this assessment, particularly if the low dose of 1.5 mg MT/kg/day identified as being an effective dose *in vivo* translates to the human clinical context. LMTM may therefore have potential as a disease-modifying treatment for synucleinopathies including PD and dementia with Lewy bodies which now requires to be tested in clinical trials.

## Author Contributions

KS, SF, DH, and JR designed and performed the experiments. MM performed immunohistochemistry and cell counting. KS and EG performed the statistical analyses. MD, ML, and TB performed the analytical determinations for MT^+^. JR and JS provided reagents. KS, JS, GR, VM, CH, CW, and FT conceived the project. KS, GR, CH, CW, and FT wrote the paper and all authors read and reviewed the final manuscript.

## Conflict of Interest Statement

This work was funded by TauRx Therapeutics Ltd., Singapore. TB, JS, CH, and CW declare that they are officers in TauRx Therapeutics Ltd. The other authors declare that the research was conducted in the absence of any commercial or financial relationships that could be construed as a potential conflict of interest.

## Abbreviations

AC + VC: auditory cortex and visual cortex
db-cAMP: dibutyryl cyclic AMP
DCE: dichloroethane
DpMe+MGV: deep mesencephalic and medial geniculate nuclei
h-α-Syn: human α-synuclein
Hip+S: hippocampus and subiculum
InG: intermediate grey layer of the superior colliculus
LMTM: leucomethylthioninium bis(hydromethanesulfonate)
MC: motor cortex
MT: methylthioninium
NGF: nerve growth factor
PD: Parkinson’s disease
RMC: magnocellular part of the red nucleus
SMC: primary sensorimotor cortex
SNpR: substantia nigra pars reticulata

## References

[B1] Ahsan N. Siddique I. A. Gupta S. Surolia A. (2017). A routinely used protein staining dye acts as an inhibitor of wild type and mutant alpha-synuclein aggregation and modulator of neurotoxicity. *Eur. J. Med. Chem.* 143 1174–1184. 10.1016/j.ejmech.2017.10.002 29150334

[B2] Allain H. Bentue-Ferrer D. Akwa Y. (2008). Disease-modifying drugs and Parkinson’s disease. *Prog. Neurobiol.* 84 25–39. 10.1016/j.pneurobio.2007.10.003 18037225

[B3] Bae E. J. Lee H. J. Rockenstein E. Ho D. H. Park E. B. Yang N. Y. (2012). Antibody-aided clearance of extracellular α-synuclein prevents cell-to-cell aggregate transmission. *J. Neurosci.* 32 13454–13469. 10.1523/JNEUROSCI.1292-12.2012 23015436 PMC3752153

[B4] Braak H. del Tredici K. (2016). Potential pathways of abnormal tau and α-synuclein dissemination in sporadic Alzheimer’s and Parkinson’s diseases. *Cold Spring Harb. Perspect. Biol.* 8:a023630. 10.1101/cshperspect.a023630 27580631 PMC5088528

[B5] Braak H. Del Tredici K. Rüb U. de Vos R. A. Jansen Steur E. N. Braak E. (2003). Staging of brain pathology related to sporadic Parkinson’s disease. *Neurobiol. Aging* 24 197–211. 10.1016/S0197-4580(02)00065-9 12498954

[B6] Carrell R. W. Gooptu B. (1998). Conformational changes and disease - serpins, prions and Alzheimer’s. *Curr. Opin. Struct. Biol.* 8 799–809. 10.1016/S0959-440X(98)80101-2 9914261

[B7] Congdon E. E. Wu J. W. Myeku N. Figueroa Y. H. Herman M. Marinec P. S. (2012). Methylthioninium chloride (methylene blue) induces autophagy and attenuates tauopathy in vitro and in vivo. *Autophagy* 8 609–622. 10.4161/auto.19048 22361619 PMC3405840

[B8] Danzer K. M. Kranich L. R. Ruf W. P. Cagsal-Getkin O. Winslow A. R. Zhu L. (2012). Exosomal cell-to-cell transmission of alpha synuclein oligomers. *Mol. Neurodegen.* 7:42. 10.1186/1750-1326-7-42 22920859 PMC3483256

[B9] Dauer W. Przedborski S. (2003). Parkinson’s disease: mechanisms and models. *Neuron* 39 889–909. 10.1016/S0896-6273(03)00568-3 12971891

[B10] Dehay B. Bourdenx M. Gorry P. Przedborski S. Vila M. Hunot S. (2015). Targeting α-synuclein for treatment of Parkinson’s disease: mechanistic and therapeutic considerations. *Lancet Neurol.* 14 855–866. 10.1016/S1474-4422(15)00006-X 26050140 PMC5217462

[B11] Delenclos M. Carrascal L. Jensen K. Romero-Ramos M. (2014). Immunolocalization of human alpha-synuclein in the Thy1-aSyn (“Line 61”) transgenic mouse line. *Neuroscience* 277 647–664. 10.1016/j.neuroscience.2014.07.042 25090921

[B12] Desplats P. Lee H. J. Bae E. J. Patrick C. Rockenstein E. Crews L. (2009). Inclusion formation and neuronal cell death through neuron-to-neuron transmission of α-synuclein. *Proc. Natl. Acad. Sci. U.S.A.* 106 13010–13015. 10.1073/pnas.0903691106 19651612 PMC2722313

[B13] Deusser J. Schmidt S. Ettle B. Plötz S. Huber S. Müller C. P. (2015). Serotonergic dysfunction in the A53T alpha-synuclein mouse model of Parkinson’s disease. *J. Neurochem.* 135 589–597. 10.1111/jnc.13253 26201615 PMC4943922

[B14] Frahm S. Melis V. Horsley D. Rickard J. E. Riedel G. Fadda P. (2017). α-Synuclein transgenic mice, h-α-SynL62, display α-Syn aggregation and a dopaminergic phenotype reminiscent of Parkinson’s disease. *Behav. Brain Res.* 339 153–168. 10.1016/j.bbr.2017.11.025 29180135

[B15] Franklin K. B. J. Paxinos G. (2008). *The Mouse Brain in Stereotaxic Coordinates, Compact* 3rd Edn. New York, NY: Academic Press.

[B16] Gauthier S. Feldman H. H. Schneider L. S. Wilcock G. K. Frisoni G. B. Hardlund J. H. (2016). Efficacy and safety of tau-aggregation inhibitor therapy in patients with mild or moderate Alzheimer’s disease: a randomised, controlled, double-blind, parallel-arm, phase 3 trial. *Lancet* 388 2873–2884. 10.1016/S0140-6736(16)31275-2 27863809 PMC5164296

[B17] Harrington C. R. Storey J. M. Clunas S. Harrington K. A. Horsley D. Ishaq A. (2015). Cellular models of aggregation-dependent template-directed proteolysis to characterize tau aggregation inhibitors for treatment of Alzheimer disease. *J. Biol. Chem.* 290 10862–10875. 10.1074/jbc.M114.616029 25759392 PMC4409250

[B18] Hasegawa T. Matsuzaki M. Takeda A. Kikuchi A. Akita H. Perry G. (2004). Accelerated α-synuclein aggregation after differentiation of SH-SY5Y neuroblastoma cells. *Brain Res.* 1013 51–59. 10.1016/j.brainres.2004.04.018 15196967

[B19] Hu Q. Uversky V. N. Huang M. Kang H. Xu F. Liu X. (2016). Baicalein inhibits α-synuclein oligomer formation and prevents progression of α-synuclein accumulation in a rotenone mouse model of Parkinson’s disease. *Biochim. Biophys. Acta* 1862 1883–1890. 10.1016/j.bbadis.2016.07.008 27425033

[B20] Ibrahim T. McLaurin J. (2016). α-Synuclein aggregation, seeding and inhibition by scyllo-inositol. *Biochem. Biophys. Res. Commun.* 469 529–534. 10.1016/j.bbrc.2015.12.043 26697752

[B21] Jakes R. Spillantini M. G. Goedert M. (1994). Identification of two distinct synucleins from human brain. *FEBS Lett.* 345 27–32. 10.1016/0014-5793(94)00395-5 8194594

[B22] Kahle P. J. Neumann M. Ozmen L. Muller V. Jacobsen H. Spooren W. (2002). Hyperphosphorylation and insolubility of α-synuclein in transgenic mouse oligodendrocytes. *EMBO Rep.* 3 583–588. 10.1093/embo-reports/kvf109 12034752 PMC1084143

[B23] Lai R. Y. Harrington C. R. Wischik C. M. (2016). Absence of a role for phosphorylation in the tau pathology of Alzheimer’s disease. *Biomolecules* 6:E19. 10.3390/biom6020019 27070645 PMC4919914

[B24] Lashuel H. A. Overk C. R. Oueslati A. Masliah E. (2013). The many faces of α-synuclein: from structure and toxicity to therapeutic target. *Nat. Rev. Neurosci.* 14 38–48. 10.1038/nrn3406 23254192 PMC4295774

[B25] Lee B. H. Lee M. J. Park S. Oh D. C. Elsasser S. Chen P. C. (2010). Enhancement of proteasome activity by a small-molecule inhibitor of USP14. *Nature* 467 179–184. 10.1038/nature09299 20829789 PMC2939003

[B26] Lee S. J. Desplats C. Sigurdson C. Tsigelny I. Masliah E. (2010). Cell-to-cell transmission of non-prion protein aggregates. *Nat. Rev. Neurol.* 6 702–706. 10.1038/nrneurol.2010.145 21045796 PMC4996353

[B27] Lindström V. Fagerqvist T. Nordström E. Eriksson F. Lord A. Tucker S. (2014). Immunotherapy targeting α-synuclein protofibrils reduced pathology in (Thy-1)-h[A30P] α-synuclein mice. *Neurobiol. Dis.* 69 134–143. 10.1016/j.nbd.2014.05.009 24851801

[B28] Luk K. C. Kehm V. Carroll J. Zhang B. O’Brien P. Trojanowski J. Q. (2012). Pathological α-synuclein transmission initiates Parkinson-like neurodegeneration in nontransgenic mice. *Science* 338 949–953. 10.1126/science.1227157 23161999 PMC3552321

[B29] Magen I. Fleming S. M. Zhu C. Garcia E. C. Cardiff K. M. Dinh D. (2012). Cognitive deficits in a mouse model of pre-manifest Parkinson’s disease. *Eur. J. Neurosci.* 35 870–882. 10.1111/j.1460-9568.2012.08012.x 22356593 PMC3967873

[B30] Mandler M. Valera E. Rockenstein E. Weninger H. Patrick C. Adame A. (2014). Next-generation active immunization approach for synucleinopathies: implications for Parkinson’s disease clinical trials. *Acta Neuropathol.* 127 861–879. 10.1007/s00401-014-1256-4 24525765 PMC4034750

[B31] Masliah E. Rockenstein E. Veinbergs I. Mallory M. Hashimoto M. Takeda A. (2000). Dopaminergic loss and inclusion body formation in α-synuclein mice: implications for neurodegenerative disorders. *Science* 287 1265–1269. 10.1126/science.287.5456.1265 10678833

[B32] Masuda M. Suzuki N. Taniguchi S. Oikawa T. Nonaka T. Iwatsubo T. (2006). Small molecule inhibitors of α-synuclein filament assembly. *Biochemistry* 45 6085–6094. 10.1021/bi0600749 16681381

[B33] Mbefo M. K. Fares M. B. Paleologou K. Oueslati A. Yin G. Tenreiro S. (2015). Parkinson disease mutant E46K enhances α-synuclein phosphorylation in mammalian cell lines, in yeast, and *in vivo*. *J. Biol. Chem.* 290 9412–9427. 10.1074/jbc.M114.610774 25657004 PMC4392248

[B34] Melis V. Magbagbeolu M. Rickard J. E. Horsley D. Davidson K. Harrington K. A. (2015a). Effects of oxidized and reduced forms of methylthioninium in two transgenic mouse tauopathy models. *Behav. Pharmacol.* 26 353–368. 10.1097/FBP.0000000000000133 25769090 PMC4416029

[B35] Melis V. Zabke C. Stamer K. Magbagbeolu M. Schwab K. Marschall P. (2015b). Different pathways of molecular pathophysiology underlie cognitive and motor tauopathy phenotypes in transgenic models for Alzheimer’s disease and frontotemporal lobar degeneration. *Cell Mol. Life Sci.* 72 2199–2222. 10.1007/s00018-014-1804-z 25523019 PMC4427622

[B36] Mena R. Edwards P. Perez-Olvera O. Wischik C. M. (1995). Monitoring pathological assembly of tau and beta-amyloid proteins in Alzheimer’s disease. *Acta Neuropathol.* 89 50–56. 10.1007/BF00294259 7709731

[B37] Mena R. Edwards P. C. Harrington C. R. Mukaetova-Ladinska E. B. Wischik C. M. (1996). Staging the pathological assembly of truncated tau protein into paired helical filaments in Alzheimer’s disease. *Acta Neuropathol.* 91 633–641. 10.1007/s004010050477 8781663

[B38] Nebrich G. Herrmann M. Sagi D. Klose J. Giavalisco P. (2007). High MS-compatibility of silver nitrate-stained protein spots from 2-DE gels using ZipPlates and AnchorChips for successful protein identification. *Electrophoresis* 28 1607–1614. 10.1002/elps.200600656 17447244

[B39] Partridge K. A. Johannessen A. Tauler A. Pryme I. F. Hesketh J. E. (1999). Competition between the signal sequence and a 3’UTR localisation signal during redirection of beta-globin mRNA to the endoplasmic reticulum: implications for biotechnology. *Cytotechnology* 30 37–47. 10.1023/A:1008079901508 19003354 PMC3449937

[B40] Peter C. Hongwan D. Kupfer A. Lauterburg B. H. (2000). Pharmacokinetics and organ distribution of intravenous and oral methylene blue. *Eur. J. Clin. Pharmacol.* 56 247–250. 10.1007/s002280000124 10952480

[B41] Piltonen M. Savolainen M. Patrikainen S. Baekelandt V. Myöhänen T. T. Männistö P. T. (2013). Comparison of motor performance, brain biochemistry and histology of two A30P α-synuclein transgenic mouse strains. *Neuroscience* 231 157–168. 10.1016/j.neuroscience.2012.11.045 23219665

[B42] R Development Core Team (2004). *R: A Language and Environment for Statistical Computing*. Vienna: R Foundation for Statistical Computing. Available at: http://www.r-project.org [accessed December 28, 2017].

[B43] Rockenstein E. Mallory M. Hashimoto M. Song D. Shults C. W. Lang I. (2002). Differential neuropathological alterations in transgenic mice expressing α-synuclein from the platelet-derived growth factor and Thy-1 promoters. *J. Neurosci. Res.* 68 568–578. 10.1002/jnr.10231 12111846

[B44] Sivanesam K. Byrne A. Bisaglia M. Bubacco L. Andersen N. (2015). Binding interactions of agents that alter α-synuclein aggregation. *RSC Adv.* 5 11577–11590. 10.1039/C5RA00325C 25705374 PMC4332700

[B45] Sommer B. Barbieri S. Hofele K. Wiederhold K. Probst A. Mistl C. (2000). Mouse models of α-synucleinopathy and Lewy pathology. *Exp. Gerontol.* 35 1389–1403. 10.1016/S0531-5565(00)00181-9 11113617

[B46] Taniguchi S. Suzuki N. Masuda M. Hisanaga S. Iwatsubo T. Goedert M. (2005). Inhibition of heparin-induced tau filament formation by phenothiazines, polyphenols, and porphyrins. *J. Biol. Chem.* 280 7614–7623. 10.1074/jbc.M408714200 15611092

[B47] Tatenhorst L. Eckermann K. Dambeck V. Fonseca-Ornelas L. Walle H. Lopes da Fonseca T. (2016). Fasudil attenuates aggregation of α-synuclein in models of Parkinson’s disease. *Acta Neuropathol. Commun.* 4 39. 10.1186/s40478-016-0310-y 27101974 PMC4840958

[B48] Tofaris G. K. Garcia Reitböck P Humby T. Lambourne S. L. O’Connell M. Ghetti B. (2006). Pathological changes in dopaminergic nerve cells of the substantia nigra and olfactory bulb in mice transgenic for truncated human α-synuclein(1-120): implications for Lewy body disorders. *J. Neurosci.* 26 3942–3950. 10.1523/JNEUROSCI.4965-05.2006 16611810 PMC6673887

[B49] Tran H. T. Chung C. H. Iba M. Zhang B. Trojanowski J. Q. Luk K. C. (2014). α-Synuclein immunotherapy blocks uptake and templated propagation of misfolded α-synuclein and neurodegeneration. *Cell Rep.* 7 2054–2065. 10.1016/j.celrep.2014.05.033 24931606 PMC4410967

[B50] Uversky V. N. (2003). A protein-chameleon: conformational plasticity of α-synuclein, a disordered protein involved in neurodegenerative disorders. *J. Biomol. Struct. Dyn.* 21 211–234. 10.1080/07391102.2003.10506918 12956606

[B51] Uversky V. N. Fink A. L. (2004). Conformational constraints for amyloid fibrillation: the importance of being unfolded. *Biochim. Biophys. Acta* 1698 131–153. 10.1016/j.bbapap.2003.12.008 15134647

[B52] van der Putten H Wiederhold K. H. Probst A. Barbieri S. Mistl C. Danner S. (2000). Neuropathology in mice expressing human α-synuclein. *J. Neurosci.* 20 6021–6029. 10934251 10.1523/JNEUROSCI.20-16-06021.2000 PMC6772584

[B53] Velayudhan L. Ffytche D. Ballard C. Aarsland D. (2017). New therapeutic strategies for Lewy body dementias. *Curr. Neurol. Neurosci. Rep.* 17:68. 10.1007/s11910-017-0778-2 28741230

[B54] Wagner J. Ryazanov S. Leonov A. Levin J. Shi S. Schmidt F. (2013). Anle138b: a novel oligomer modulator for disease-modifying therapy of neurodegenerative diseases such as prion and Parkinson’s disease. *Acta Neuropathol.* 125 795–813. 10.1007/s00401-013-1114-9 23604588 PMC3661926

[B55] Wilcock G. K. Gauthier S. Frisoni G. B. Jia J. Hardlund J. H. Moebius H. J. (2018). Potential of low dose leuco-methylthioninium bis(hydromethanesulphonate) (LMTM) monotherapy for treatment of mild Alzheimer’s disease: cohort analysis as modified primary outcome in a phase 3 clinical trial. *J. Alzheimers Dis.* 61 435–457. 10.3233/JAD-170560 29154277 PMC5734125

[B56] Wischik C. M. Edwards P. C. Lai R. Y. K. Roth M. Harrington C. R. (1996). Selective inhibition of Alzheimer disease-like tau aggregation by phenothiazines. *Proc. Natl. Acad. Sci. U.S.A.* 93 11213–11218. 10.1073/pnas.93.20.11213 8855335 PMC38310

[B57] Wischik C. M. Staff R. T. Wischik D. J. Bentham P. Murray A. D. Storey J. M. (2015). Tau aggregation inhibitor therapy: an exploratory phase 2 study in mild or moderate Alzheimer’s disease. *J. Alzheimers Dis.* 44 705–720. 10.3233/JAD-142874 25550228

[B58] Zhang X. Rojas J. C. Gonzalez-Lima F. (2006). Methylene blue prevents neurodegeneration caused by rotenone in the retina. *Neurotox. Res.* 9 47–57. 10.1007/BF03033307 16464752

[B59] Zhu M. Rajamani S. Kaylor J. Han S. Zhou F. Fink A. L. (2004). The flavonoid baicalein inhibits fibrillation of α-synuclein and disaggregates existing fibrils. *J. Biol. Chem.* 279 26846–26857. 10.1074/jbc.M403129200 15096521

